# Skin Diseases and Syphilis

**Published:** 1895-04

**Authors:** 


### *SELECTIONS AND ABSTRACTS.*

---

### SKIN DISEASES AND SYPHILIS.

EDITED BY DR. M. B. HUTCHINS.

### EPITHELIOMA OF THE PENIS.

Dr. Edward Martin of Philadelphia, has a paper under this title in the *Journal of Cutaneous Diseases and Genito-Urinary Diseases*, March, 1895. He gives the history of the case of a man of sixty-two. An aunt had cancer of the stomach. Patient never had syphilis. Eight years previous a small, red spot in prepuce, inner surface. Hard, but only slight pain. Gradually spread backward, with increase of pain, for five years, the ulcer becoming larger and harder. Lesion then cauterized; patient using nitrate of silver stick six times in three years; following with a simple salve. Apparent healing followed each time, but breaking down soon occurred, showing a central hard nodule. When seen by Dr. Martin it was three months since last treatment.

Upon examination, phimosis, slight œdema of prepuce, prepuce and glans adherent above where a thumb-end sized hard, irregular nodule was perceptible, extending one-fourth inch behind corona. Strong retraction of prepuce exposed a hard, projecting mass like indolent granulation tissue. No perceptible enlargement of dorsal lymph vessels, nor inguinal lymphatic glands. Sharp, lancinating pains and bloody discharge from preputial orifice for the last six months. No history of sore or injury preceding the disease.

He then briefly mentions the operation done. A cut representing the microscopic appearances accompanies the article. The microscope showed perfectly healthy tissue a quarter of an inch behind the perceptible enlargement. No appearance of disease in the stump or inguinal lymphatics nine months afterwards, the time of the writer's report.

In six of the seven cases which he had seen, phimosis preceded the cancer's development. McFarland had found in literature eight cases of contagion from wife to husband. Other cases reported pointed to direct contagion. "A limited epidemic" of cancer has been reported (Fiessinger and Guelliot). As to syphilis (chancre) predisposing to epithelioma he thinks the scar only furnished a focus of lessened resistance.

As to prognosis, it is good where promptly and thoroughly treated in the "precancerous stage," that is during its first development. When typically developed, the prognosis is less favorable, the inguinal glands being commonly involved, and even the penile infiltration is so extensive that local recurrence takes place after the ordinary operation. The lymphatic glands may be involved before they are perceptibly enlarged or simply inflamed, the inflammation disappearing after thorough removal of the cancer.

"Visceral metastases are extremely rare."

Untreated, the disease is fatal, but may last ten years or more. Operation prolongs life even when all diseased tissue cannot be removed.

Absence of recurrence for various periods in years have been reported; but, as the writer says, no accurate knowledge of the percentage of recurrence can be obtained, as there is a tendency only to report favorable cases. He very justly believes that a careful microscopic examination of the tissues removed will show the chances of return (*i. e.*, whether all the disease has been removed).

In the "precancerous" stage it may suffice to thoroughly cauterize, or excise and cauterize. Upon signs of recurrence, amputate; if still not thorough, extirpate—all inguinal glands also in typically developed cases.

The design of the paper was to call especial attention to—

(1) A positive denial of the contagion of epithelioma of the penis is unjustifiable.

(2) Amputation being required, the inguinal glands of both sides should be dissected out, even though not appreciably enlarged.

---

### HARD CHANCRE OF THE EYELIDS.

Panas (*Journ. de Medicine et de Chirurgic Pratiques*, December 10, '93) says chancre of the eyelids occurs more frequently than one perceives, but it is easily confused with malignant pustule, cancroid, or lupus. Its usual site is the canthus, involving also both lids. Related glands of the face are involved early. The treatment must be energetic, with hypodermic injections of biniodide of mercury; internally, iodide of potassium. The ulcer must not be cauterized, as ectropium easily occurs.—*Monatschefte für Prak. Derm., XIX, No. II.*

